# Niche Partitioning Promotes Coexistence: Habitat Suitability and Spatial Overlap of Three Sympatric Ungulates in a Subtropical Mountain Reserve

**DOI:** 10.1002/ece3.73248

**Published:** 2026-03-20

**Authors:** Jie Yao, Xueqin Hu, Jun Tian, Feiyan Lv, Ruijie Yang, Zhiqiang Huang, Jiancheng Zhai

**Affiliations:** ^1^ School of Earth and Planetary Sciences East China University of Technology Nanchang China; ^2^ Institute of Natural Reserve Planning and Research East China University of Technology Nanchang China

**Keywords:** conservation management, habitat suitability, MaxEnt, niche overlap, ungulates

## Abstract

Understanding species–environment relationships and habitat overlap among sympatric ungulates is crucial for biodiversity conservation in mountainous ecosystems. Using a three‐year camera‐trap dataset (2021–2024) from Tongboshan National Nature Reserve, Jiangxi, China, we applied MaxEnt modeling to evaluate habitat suitability and niche overlap of Black muntjac ( *Muntiacus crinifrons* ), Reeves's muntjac ( *Muntiacus reevesi* ), and Wild boar ( *Sus scrofa* ). After spatial filtering, 103 independent occurrence records were analyzed against 12 environmental predictors representing topography, vegetation, soil, water, and anthropogenic disturbance. All models performed well (AUC > 0.90), with vegetation and topography contributing most to species distributions. Black muntjac exhibited a narrow spatial niche breadth, being strongly associated with mid‐ to high‐elevation forests and close proximity to water sources, whereas Reeves's muntjac showed broad ecological flexibility, adapting to coniferous forests and moderately disturbed landscapes. Wild boar demonstrated generalist strategies, favoring forest edges and bamboo stands. Predicted suitable habitats covered 16.75%, 41.54%, and 31.17% of the reserve for Black muntjac, Reeves's muntjac, and Wild boar, respectively. Niche overlap analysis revealed the highest overlap between Reeves's muntjac and Wild boar (*D* = 0.822; *I* = 0.969), while Black muntjac showed limited overlap with either species. Species‐specific habitat preferences and spatial partitioning emerge as key mechanisms enabling coexistence. We recommend prioritizing the maintenance of closed‐canopy forest habitats for Black muntjac as the most critical conservation strategy, alongside enhancing forest heterogeneity in overlap zones and establishing ecological corridors to improve landscape connectivity. Our study provides quantitative evidence to guide adaptive management and support multi‐species conservation in subtropical reserves.

## Graphical abstract

This study assessed the habitat suitability and spatial niche differentiation of three sympatric ungulates—Black muntjac, Reeves's muntjac, and Wild boar—in the Tongbo Mountain National Nature Reserve, China. Using MaxEnt modeling and niche overlap analysis, we found distinct habitat preferences and hierarchical niche segregation that facilitate species coexistence and provide quantitative guidance for multi‐species conservation.

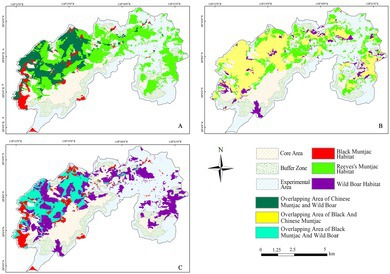

## 1 Introduction

Ungulates are key functional groups in forest and grassland ecosystems, widely recognized as major drivers of community structure and ecosystem functioning due to their multiple ecological roles in vegetation regulation, seed dispersal, and soil disturbance (Hobbs 1996; Augustine and McNaughton 1998; Albert et al. 2015). Through foraging activities, they influence plant composition and dynamics, modify microhabitat conditions (Ameztegui and Coll 2015), and play central roles in energy flow and nutrient cycling within food webs (Hanley 1982), thereby contributing indispensably to ecosystem stability and resilience. Recent studies have further highlighted that ungulates also provide important ecosystem services such as maintaining genetic resources, regulating pathogen transmission, and suppressing insect pests, with far‐reaching implications for human well‐being (Roldan et al. 2006; Reimoser 2011; Jolles and Ezenwa 2015). However, under accelerating global climate change and increasing anthropogenic disturbances, many wild ungulate species are facing multiple threats, including habitat loss, population fragmentation, and poaching (Ito et al. 2013; Said et al. 2016). Assessments by the International Union for Conservation of Nature (IUCN) indicate that their populations are generally in decline, making their conservation and management a core issue in global biodiversity strategies. Despite their ecological importance, conservation planning for ungulates has largely focused on single‐species approaches, with limited consideration of spatial overlap and habitat partitioning among sympatric species within shared landscapes. This knowledge gap is particularly critical in biodiverse mountain ecosystems, where species coexistence is shaped by complex environmental gradients and spatial constraints.

Understanding the ecological relationships among coexisting species is crucial for developing effective conservation measures. In ungulate communities, where species often overlap extensively in habitat use, niche theory provides a powerful framework for examining spatial mechanisms of coexistence and resource partitioning (Vandermeer 1972). Recent research on sympatric ungulates suggests that spatial niche differentiation is a key pathway for minimizing competition and facilitating coexistence (Aarssen 1984). For example, in typical montane ecosystems such as Minya Konka and Mount Fanjing, studies have revealed that species including the Tufted deer ( *Elaphodus cephalophus* ), Reeves's muntjac ( *Muntiacus reevesi* ), Wild boar ( *Sus scrofa* ), and Mainland serow ( *Capricornis milneedwardsii* ) exhibit pronounced differences in habitat selection and spatial use (Qiao et al. 2022; Xiang et al. 2024). Even under resource‐limited conditions, these species achieve long‐term coexistence through varying degrees of niche overlap and differentiation. Such findings underscore that spatial niche analysis not only identifies potential competitive and complementary relationships, but also provides operational insights for multi‐species conservation and habitat management. Nevertheless, how such spatial overlap and niche segregation are quantitatively structured, and how these patterns translate into conservation‐relevant outcomes for threatened specialists, remains insufficiently understood.

Tongboshan National Nature Reserve, Jiangxi, China, is located at the junction of the Wuyi, Xianxia, and Huaiyu mountain ranges in eastern China. It serves as a critical ecological corridor in eastern China, playing a strategic role in maintaining regional connectivity and facilitating species dispersal. The reserve is characterized by complex mountainous terrain and diverse vegetation types, which typify the mid‐subtropical montane ecosystem and provide suitable habitats and breeding conditions for a wide range of wildlife (Zhang et al. 2013). Among the ungulates distributed in this area, the Black muntjac ( *Muntiacus crinifrons* ) is endemic to China and listed as a national first‐class protected species. It is classified as “Vulnerable” (VU) by the IUCN owing to its highly restricted geographic range, strong habitat specialization, and high sensitivity to habitat disturbance, with its distribution limited to a few mountainous regions of eastern China (Ho‐Gee and He‐Lin 1984). Coexisting with this species are Reeves's muntjac and Wild boar, which represent contrasting ecological strategies. The former is a broadly distributed generalist with high adaptability, while the latter is an opportunistic forager capable of exploiting fragmented and edge habitats, occupying key positions in a wide range of ecosystems (Yang et al. 1997; Piattoni et al. 2016). The marked contrast in ecological strategies among these three sympatric ungulates makes Tongboshan an ideal natural system for examining how spatial habitat overlap and niche differentiation facilitate coexistence, particularly for endemic and threatened species under potential competitive pressure.

Although previous studies have examined aspects of Black muntjac biology, such as reproductive behavior, habitat use, and genetic structure (Hemami et al. 2004; Wu et al. 2006; Houji 2011), systematic investigations into its spatial niche relationships with Reeves's muntjac and Wild boar remain scarce. In particular, explicit, quantitative analyses of spatial habitat overlap, niche differentiation, and their implications for coexistence and conservation are still lacking. Based on long‐term camera‐trap monitoring in Tongboshan National Nature Reserve, this study focuses on three representative ungulate species (Black muntjac, Reeves's muntjac, and Wild boar) to construct comparative niche analyses and spatial distribution models. We hypothesize that sympatric ungulates exhibit a hierarchical pattern of spatial niche breadth and overlap, whereby generalist species occupy broader suitable habitats, while specialist species are constrained to narrower, high‐quality habitat subsets. Specifically, the objectives are to: (1) identify the potential distribution patterns and habitat preferences of the three species within the reserve; (2) reveal their patterns of spatial niche overlap and differentiation; and (3) assess the potential competitive pressures and conservation risks faced by the Black muntjac. The findings not only provide theoretical and practical guidance for targeted conservation of this endemic threatened species and the scientific management of protected areas, but also offer valuable insights into mechanisms of multi‐species coexistence in mid‐subtropical montane ecosystems.

## 2 Materials and Methods

### 2.1 Study Area

**FIGURE 1:**
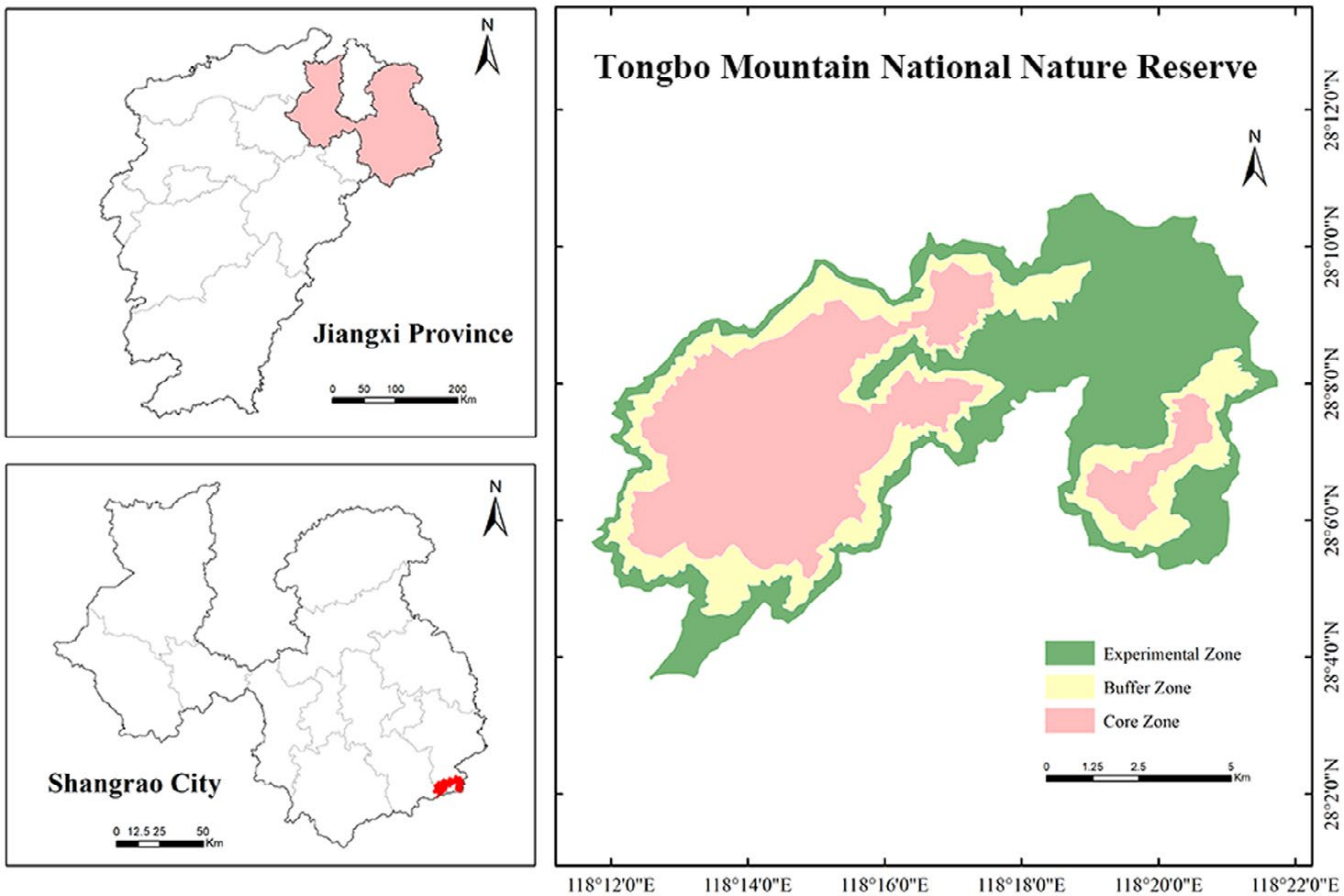
Location and overview of the Tongboshan National Nature Reserve.

Tongboshan National Nature Reserve is located in southern Guangfeng District, Shangrao City, Jiangxi Province, at the transitional zone where the Wuyi, Xianxia, and Huaiyu mountain ranges converge (Figure 1). The reserve covers an area of approximately 108 km^2^, with geographic coordinates ranging from 118°11′42″ E to 118°21′47″ E and 28°03′39″ N to 28°10′45″ N. The landscape is dominated by mid‐ to high‐montane terrain characterized by steep relief, with elevations ranging from 350 to 1450 m and slopes typically between 40° and 70°. The area is dissected by dense streams and deep valleys, forming a typical subtropical montane forest mosaic with pronounced elevational and topographic heterogeneity (Shan et al. 2014).

The region has a subtropical monsoon climate, with a mean annual temperature of 11.0°C–17.1°C and annual precipitation between 1739.4 and 1892.7 mm. The climate is warm and humid, with rainfall and temperature peaking in the same season. Forest cover reaches 94.6%, and the major vegetation types include evergreen broad‐leaved forest, coniferous and broad‐leaved mixed forest, evergreen coniferous forest, and *Phyllostachys pubescens* forests, together providing structurally complex habitats (Zhang et al. 2013).

Owing to its complex terrain and rich ecological resources, the reserve harbors high wildlife diversity. Historical monitoring and recent surveys have documented 337 species of vertebrates, including 44 mammals, 198 birds, 73 amphibians and reptiles, and 22 fishes. The reserve supports 37 nationally protected wildlife species in China, including six Class I species (e.g., Black muntjac and clouded leopard ( *Neofelis nebulosa* )) and four Class II species (e.g., rhesus macaque ( *Macaca mulatta* ) and mainland serow). These records highlight the reserve's significance as a core unit for regional biodiversity conservation. Tongboshan plays an irreplaceable role in maintaining the connectivity of southern forest ecosystems and supporting the survival and evolution of endemic and threatened species. Moreover, it provides an ideal natural setting for investigating niche differentiation, habitat selection, and coexistence mechanisms among wild ungulates.

### 2.2 Camera‐Trap Survey and Occurrence Data

Species occurrence data were obtained from a continuous camera‐trap monitoring program conducted in Tongboshan National Nature Reserve between June 2021 and August 2024. A total of 161 infrared cameras (model: E3H, Shenzhen Ereagle Technology Co. Ltd.) were deployed across the core, buffer, and experimental zones of the reserve. Camera stations were established based on topographic conditions, accessibility, and signs of wildlife activity. To ensure representative coverage and spatial independence, inter‐station spacing was maintained at 500–1000 m. Over the study period, cameras accumulated approximately 1155 effective trap‐days. All images were manually screened to remove false triggers, empty frames, and blurred records, and independent detection events were extracted as occurrence samples. Target species for this study included Black muntjac, Reeves's muntjac, and Wild boar.

**FIGURE 2:**
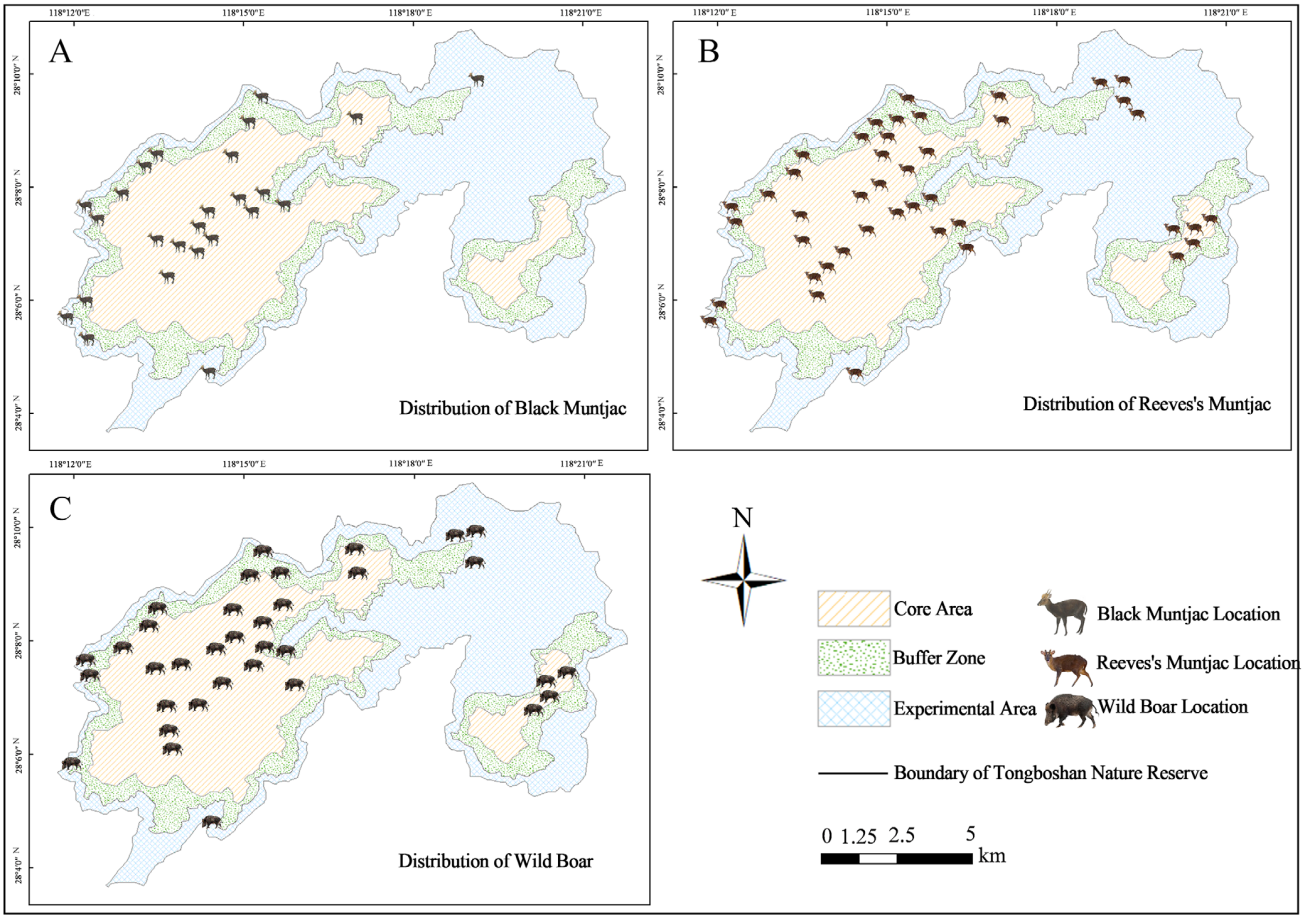
Distribution records of the three ungulate species after data screening.

To minimize the influence of spatial autocorrelation on model predictions (Legendre 1993), we selected a spatial thinning distance informed by species‐specific movement ecology. Reported home range sizes suggest a radius of approximately 560–690 m for Black muntjac (1–1.5 km^2^), around 480 m for Reeves's muntjac, and equal or greater spatial use for Wild boar. Accordingly, a conservative 500 m buffer was applied to remove clustered duplicate records (McCullough et al. 2000). After excluding overlapping and outlier locations, a total of 103 spatially independent occurrence points were retained, comprising 25 for Black muntjac, 43 for Reeves's muntjac, and 35 for Wild boar (Figure 2). These records were used as input data for species distribution modeling with the maximum entropy model (MaxEnt).

### 2.3 Environmental Predictors and Preprocessing

To improve the predictive accuracy of the MaxEnt model and reduce the influence of redundant variables, we adopted a multi‐step procedure for environmental variable selection, integrating ecological theory, species‐specific traits, and modeling practice (McArthur and Baron 1983; Chenyang et al. 2023).

First, based on previous studies on ungulate distributions (Borowik et al. 2013; Desforges et al. 2021; Johnson et al. 2002; Santos et al. 2018), we initially selected 17 environmental variables representing four categories, namely topography, vegetation, soil and water factors, and anthropogenic disturbance. These environmental variables may influence habitat selection and species distribution in Tongboshan National Nature Reserve (Table 1). Topographic data were derived from a 30 m resolution digital elevation model (DEM) available from the Chinese Geospatial Data Cloud (https://www.gscloud.cn). Vegetation and soil variables were obtained from the second forest resource inventory of the reserve, while water‐related variables (e.g., distance to streams and water bodies) and anthropogenic disturbance variables were extracted from the latest vector layers provided by Tongboshan National Nature Reserve Administration.

**FIGURE 3:**
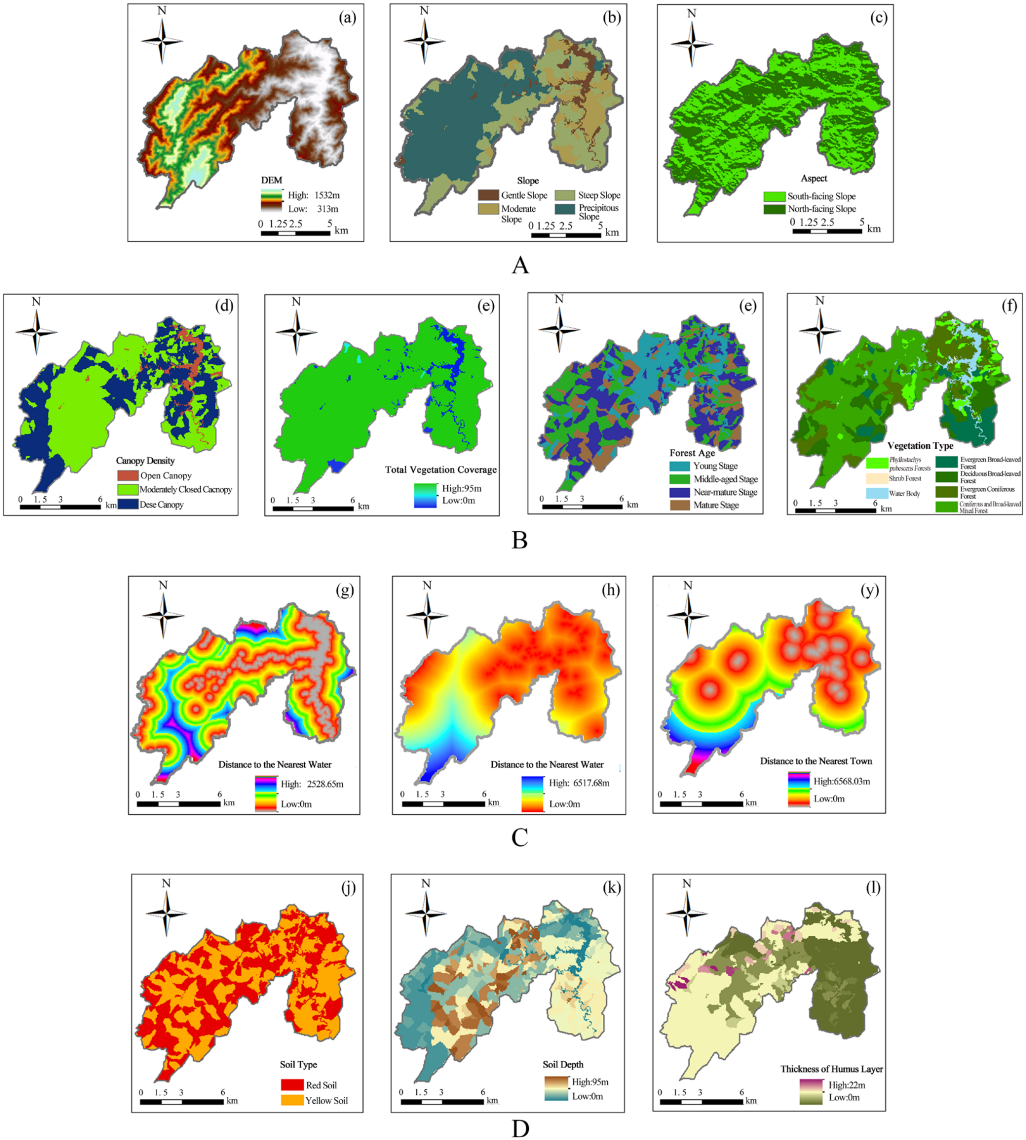
Environmental predictor variables used for habitat modeling: (A) Topographic variables (elevation, slope, and aspect); (B) vegetation variables (vegetation type, canopy closure, total vegetation cover, and stand age); (C) hydrological and anthropogenic variables (distance to water sources, settlements, and roads); (D) soil variables (soil type, soil depth, and humus layer thickness).

All environmental variables were preprocessed in ArcGIS 10.8 (Figure 3). All raster layers were projected to a unified coordinate system (CGCS2000), clipped to the reserve boundary, resampled to a 30 × 30 m resolution, and checked for data integrity (value ranges and NoData cells) using raster calculator and statistical tools. The resulting dataset was converted into ASCII raster format for subsequent use in MaxEnt modeling.

**TABLE 1:** Description and coding of environmental variables used in habitat modeling for Tongboshan National Nature Reserve.

Categories	Variables	Code	Data type	Range/content
Topographic	DEM (m)	dem	Continuous	317–1513
	Slope (°)	slope	Categorical	Gentle/Moderate/Steep/Precipitous
	Aspect	aspect	Categorical	South‐facing/North‐facing
Vegetation	Distance to the Evergreen Broad‐leaved Forest (m)	da	Continuous	0–5147
	Distance to the Evergreen and Deciduous Broad‐leaved Mixed Forest (m)	db	Continuous	0–2240
	Distance to the Evergreen Coniferous Forest (m)	dc	Continuous	0–2519
	Distance to the Coniferous and Broad‐leaved Mixed Forest (m)	dd	Continuous	0–2349
	Distance to the *Phyllostachys pubescens* forests	de	Continuous	0–4089
	Canopy density	ybd	Categorical	Open/Moderately canopy/Dense
	Total vegetation coverage (%)	tvc	Continuous	0–95
	Forest age	fa	Categorical	Young/Middle‐aged/Near‐mature/Mature
Soil	Soil type	st	Categorical	Red soil/Yellow soil
	Soil depth (cm)	sd	Continuous	0–95
	Thickness of humus layer (cm)	thl	Continuous	0–22
Water sources and Anthropogenic disturbance	Distance to the Nearest Water (m)	dw	Continuous	0–2489
	Distance to the Nearest Road (m)	dr	Continuous	0–6437
	Distance to the Nearest Town (m)	dt	Continuous	0–6540

To further minimize the effect of irrelevant variables, environmental data were standardized using ENMTools 1.3 (Warren et al. 2010). Variables contributing less than 1% to model gain were excluded, as such variables typically provide limited explanatory power while increasing model complexity. Pairwise Pearson correlation analysis was then conducted, and when two variables were highly correlated (|r| > 0.8), model performance was compared using the area under the receiver operating characteristic curve (AUC). The variable with the lower AUC was removed, while the other was retained (Bradley 1997). AUC was selected because it is threshold‐independent and particularly suitable for presence‐only models, allowing consistent comparison of model discrimination ability across alternative predictor sets.

**FIGURE 4:**
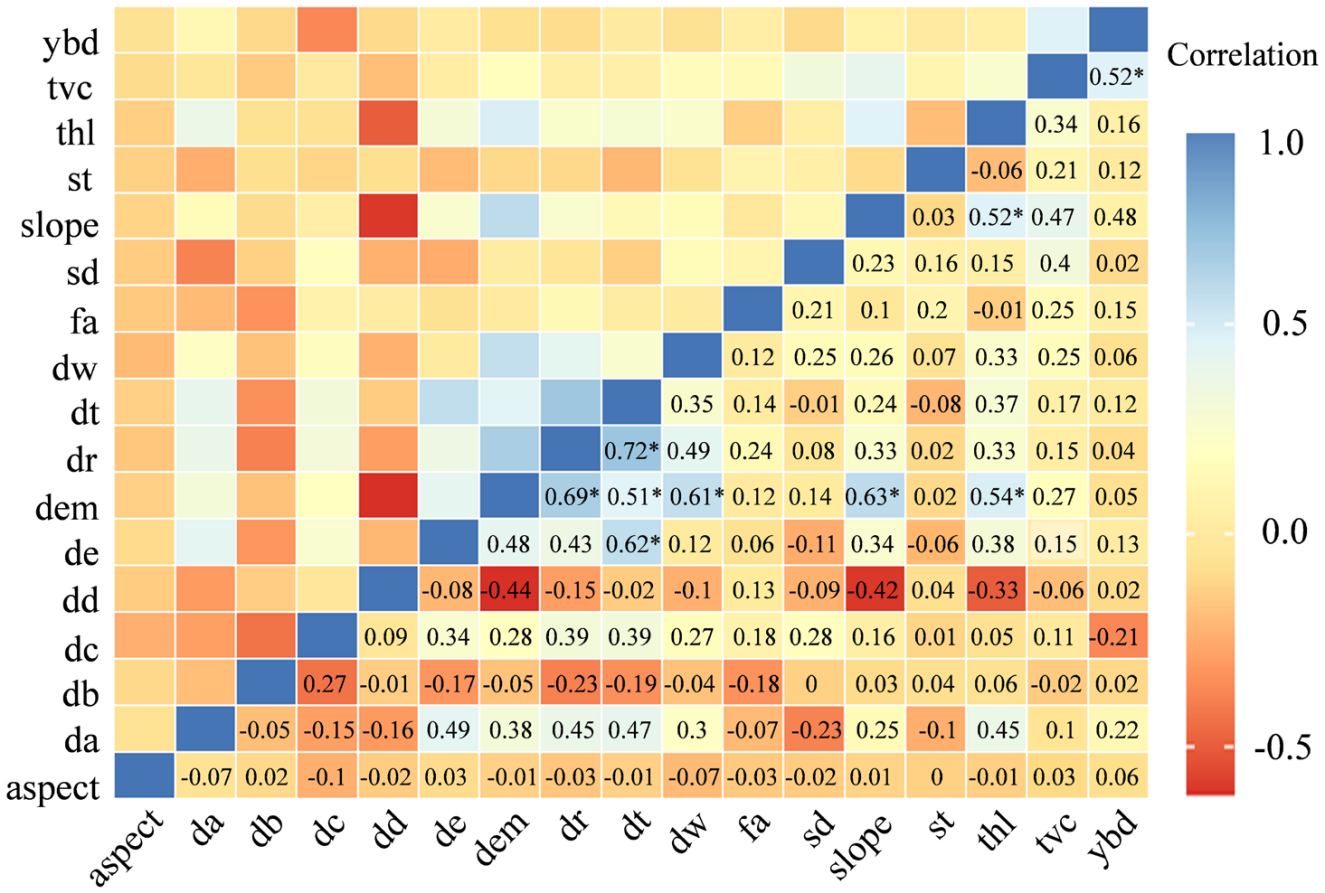
Pearson correlation matrix of environmental variables.

The final set of 12 environmental predictors was identical for all three species to ensure comparability in habitat suitability estimation and spatial niche overlap analyses. These predictors encompassed topographic, vegetation, soil, water‐related, and anthropogenic disturbance variables and were used consistently in habitat suitability modeling for Black muntjac, Reeves's muntjac, and Wild boar (Figure 4).

### 2.4 Species Distribution Modeling With MaxEnt

The MaxEnt is a machine learning algorithm widely used in species distribution modeling, particularly suitable for presence‐only datasets with limited sample sizes (Phillips and Dudík 2008). By maximizing entropy, the model establishes nonlinear relationships between species occurrence and environmental variables, thereby predicting habitat suitability across the study area (Elith et al. 2011). MaxEnt has been extensively applied in endangered species conservation, habitat suitability assessment, and climate change impact studies (Kumar and Stohlgren 2009; Khanum et al. 2013; Ab Lah et al. 2021). In this study, we used MaxEnt version 3.4.4 to model the potential habitat suitability of Black muntjac, Reeves's muntjac, and Wild boar.

We enabled the Bootstrap option and activated the random seed parameter to enhance model stability and reproducibility. Response curves were generated to visualize species–environment relationships, and Jackknife tests were used to quantify the independent explanatory power of each predictor. Model outputs were generated as continuous raster layers in logistic format, ranging from 0 to 1. Default MaxEnt feature classes and the default regularization multiplier (RM = 1) were retained. To reduce stochastic error, each species model was run 10 times independently, and results were averaged across replicates.

To assess model robustness and sensitivity, we compared AUC values across replicate runs and examined the consistency of variable importance rankings and response curves. Stable performance across bootstrap replicates was interpreted as evidence of low model sensitivity to sampling variation. The performance of the models was evaluated using the area under the receiver operating characteristic curve (AUC) (Muschelli III 2020). According to conventional thresholds, an AUC value of 0.8–0.9 indicates good predictive performance, while AUC > 0.9 is considered excellent.

### 2.5 Quantifying Habitat Overlap Among Species

To assess the spatial overlap among the three ungulate species, we conducted niche overlap analyses using ENMTools v1.3 (Warren et al. 2019). First, the maximum training sensitivity plus specificity logistic threshold was applied to determine the optimal cutoff (Coughlin et al. 1992), converting the continuous probability outputs (0–1) of MaxEnt into binary maps of suitable habitat (1) and unsuitable habitat (0). These thresholded distribution maps were then imported into ENMTools to quantify pairwise niche overlap among species.

Niche overlap between each pair of species was quantified using Schoener's D and the Hellinger‐based I indices (Mukherjee et al. 2021; Yin et al. 2021).

(1) $$D=1-\frac{1}{2}\sum_{i=1}^{n}\left|P_{\mathrm{iA}}-P_{\mathrm{iB}}\right|$$

(2) $$I=\sum_{i=1}^{n}\sqrt{P_{\mathrm{iA}}\times P_{\mathrm{iB}}}$$

where: $P_{\mathrm{iA}}$ and $P_{\mathrm{iB}}$ are the normalized habitat suitability scores for species A and B, respectively, in grid cell i; n is the total number of grid cells across the study area.

The D index measures the absolute differences between probability distributions and reflects the degree of resource‐use overlap, while the *I* index, derived from Hellinger distance, captures the similarity between spatial probability distributions. Both indices range from 0 (no overlap) to 1 (complete overlap), with higher values indicating greater spatial niche similarity. Because D emphasizes resource utilization and I emphasizes distributional similarity, the two metrics are complementary.

In addition, spatial overlay analyses were performed in ArcGIS 10.8 to extract areas of suitable habitat overlap among species. We quantified the proportion of overlapping habitat relative to each species' total predicted range and identified potential competition zones, coexistence core areas, and key ecological corridors. These results provide spatially explicit insights to support reserve management and multi‐species conservation planning.

## 3 Results

### 3.1 Model Performance and Habitat Responses

**FIGURE 5:**
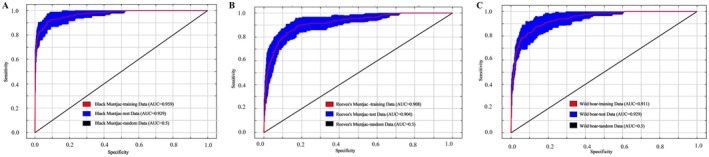
Performance evaluation of the MaxEnt models for the three ungulate species.

Wildlife spatial distributions are generally adaptive responses to complex environmental gradients, with habitat selection jointly influenced by topography, vegetation, water availability, and human disturbance. Using MaxEnt modeling, we predicted the potential suitable habitats of Black muntjac, Reeves's muntjac, and Wild boar in Tongboshan National Nature Reserve and evaluated the relative contributions of environmental variables. All three models showed strong predictive performance, with mean AUC values (averaged across 10 replicate runs) consistently exceeding 0.90, indicating high discriminatory capacity and robustness (Figure 5). Among the three species, the Black muntjac model exhibited the highest overall performance, followed by Wild boar and Reeves's muntjac (Figure 5A–C).

**FIGURE 6:**
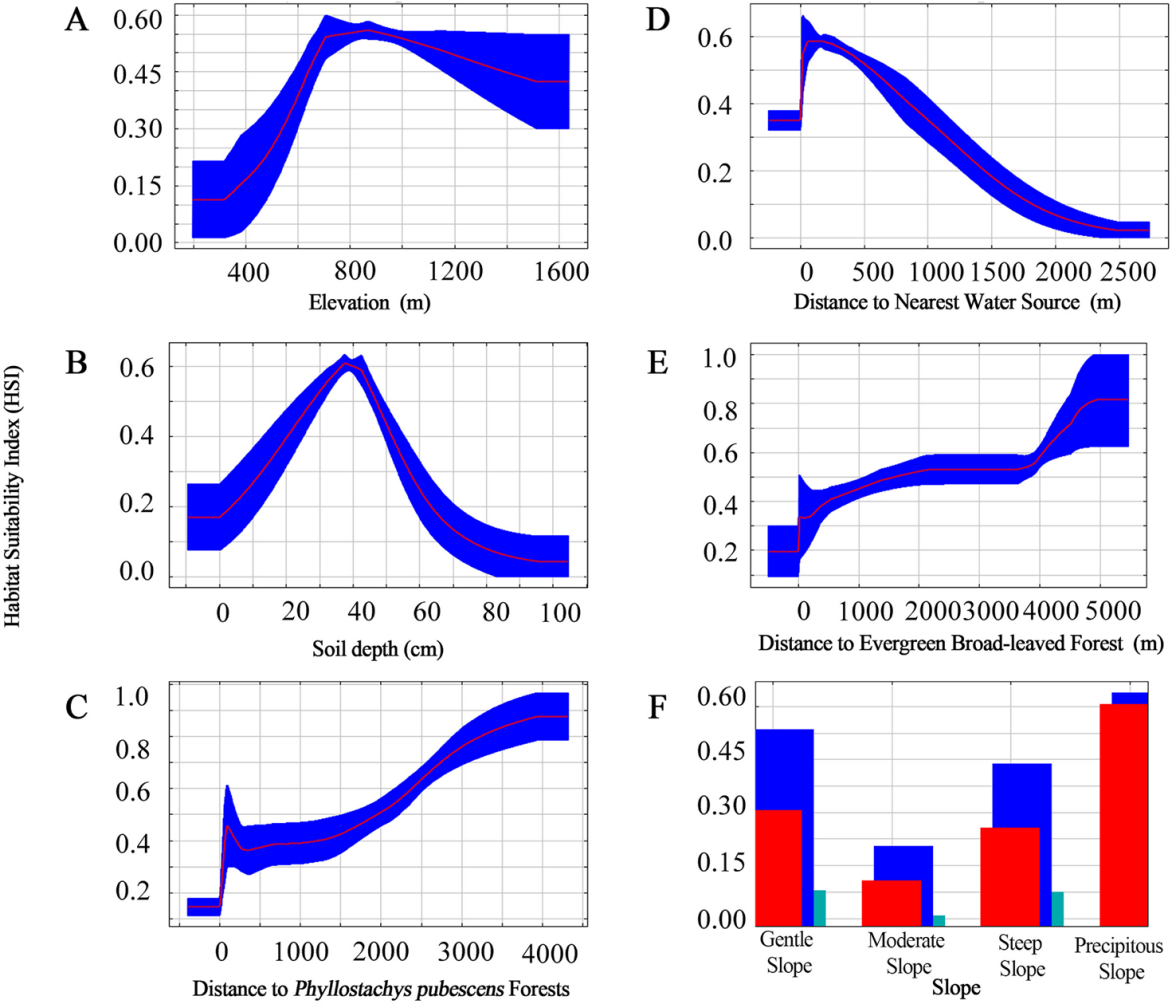
Response curves of key environmental variables affecting habitat suitability of *Muntiacus crinifrons* . Red lines represent the mean response across 10 replicates. For continuous variables, blue shading indicates mean ± SD; for categorical variables, blue and green bars represent +SD and –SD, respectively.

For Black muntjac, the mean training AUC was 0.959 and the mean test AUC was 0.929, representing the highest model performance among the three focal species (Figure 5A). Among individual predictors, elevation was the single most influential variable (15.4%), followed by distance to the nearest water source (10.6%) and soil depth (10.5%), indicating a strong dependence on topographic and moisture‐related conditions (Table 2). At the variable‐group level, vegetation (35.8%) and topography (25.7%) together dominated model contributions, whereas soil properties and water‐ and disturbance‐related variables played secondary but non‐negligible roles. Response curve analysis (Figure 6) revealed a clear unimodal relationship between habitat suitability and elevation, with suitability peaking at approximately 931 m, suggesting a preference for mid‐ to high‐elevation forests. Habitat suitability increased sharply within approximately 70 m of water sources, underscoring the species' reliance on moist microhabitats. In contrast, suitability declined in areas dominated by evergreen broad‐leaved forests and *Phyllostachys pubescens* forests, while mixed and evergreen coniferous forests on moderately deep soils were consistently favored.

**TABLE 2:** Relative contributions of environmental variables to habitat suitability for *Muntiacus crinifrons*.

Variables	Contributions (%)	Importance (%)
DEM (m)	15.4	20
Distance to the Nearest Water (m)	10.6	8.1
Soil depth (cm)	10.5	2.6
Distance to the Evergreen Broad‐leaved Forest (m)	9.2	2.1
Distance to the *Phyllostachys pubescens* Forests (m)	7.9	18.3
Slope (°)	7.2	2.1
Distance to the Nearest Town (m)	7.1	14.1
Soil type	5.2	3.6
Distance to the Nearest Road (m)	4.8	7.4
Distance to the Evergreen Coniferous Forest (m)	4.8	12.2
Distance to the Evergreen and Deciduous Broad‐leaved Mixed Forest (m)	3.7	2.7
Total vegetation coverage (%)	3.4	0.8
Aspect	3.1	0.4
Forest age	2.9	1.6
Canopy density	0.4	0.3
Thickness of humus layer (cm)	0.3	0.9

**FIGURE 7:**
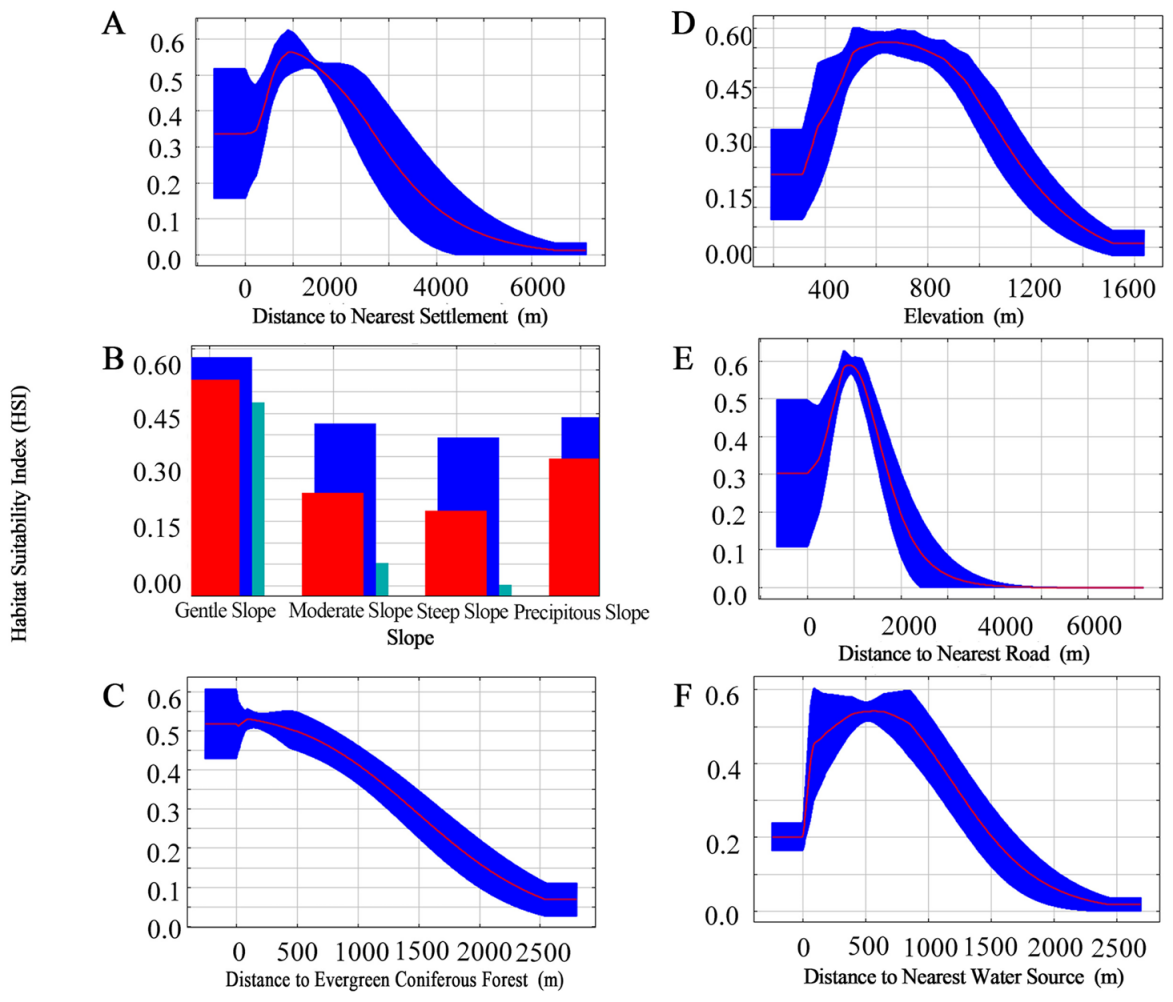
Response curves of key environmental variables affecting habitat suitability of *Muntiacus reevesi* .

For Reeves's muntjac, the MaxEnt model achieved a mean training AUC of 0.908 and a mean test AUC of 0.904, averaged across the 10 replicate runs, indicating stable and reliable predictive performance (Figure 5B). Among individual predictors, distance to the nearest settlement was the most influential variable (15.3%), followed by elevation (10.0%), slope (9.7%), and distance to the nearest road (8.3%), underscoring the combined roles of topography and anthropogenic disturbance in shaping habitat suitability (Table 3). At the categorical level, vegetation variables contributed most strongly to the model (38.3%), followed by water‐ and disturbance‐related variables (29.6%) and topographic factors (23.9%), whereas soil properties played a minor role. Response curves (Figure 7) showed that Reeves's muntjac was particularly sensitive to human disturbance, with habitat suitability peaking at intermediate distances from settlements and roads, indicating avoidance of both heavily disturbed and highly remote areas. Slope and road distance exhibited unimodal relationships with suitability, reflecting a preference for moderately sloped terrain with limited accessibility. In terms of vegetation, suitability declined with increasing distance from evergreen coniferous forests, highlighting the species' strong association with forest types characterized by relatively open understories. Compared with Black muntjac, suitability in relation to water sources increased less sharply, indicating a weaker dependence on water availability and greater ecological flexibility.

**TABLE 3:** Relative contributions of environmental variables to habitat suitability for *Muntiacus reevesi*.

Variables	Contributions (%)	Importance (%)
Distance to the Nearest Town (m)	15.3	19
DEM (m)	10	18
Slope (°)	9.7	5.5
Distance to the Nearest Road (m)	8.3	6.5
Distance to the Evergreen Coniferous Forest (m)	7	7.7
Distance to the Nearest Water (m)	6	4.9
Forest age	6	4.4
Distance to the *Phyllostachys pubescens* Forests (m)	5.6	4.6
Distance to the Evergreen Broad‐leaved Forest (m)	4.8	6.4
Distance to the Evergreen and Deciduous Broad‐leaved Mixed Forest (m)	4.3	3.2
Aspect	4.2	1.7
Total vegetation coverage (%)	3.8	2.7
Distance to the Coniferous and Broad‐leaved Mixed Forest (m)	3.7	6.3
Soil depth (cm)	3.7	5.4
Canopy density	3.1	0.5
Thickness of humus layer (cm)	2.5	2.9
Soil type	2	0.8

**FIGURE 8:**
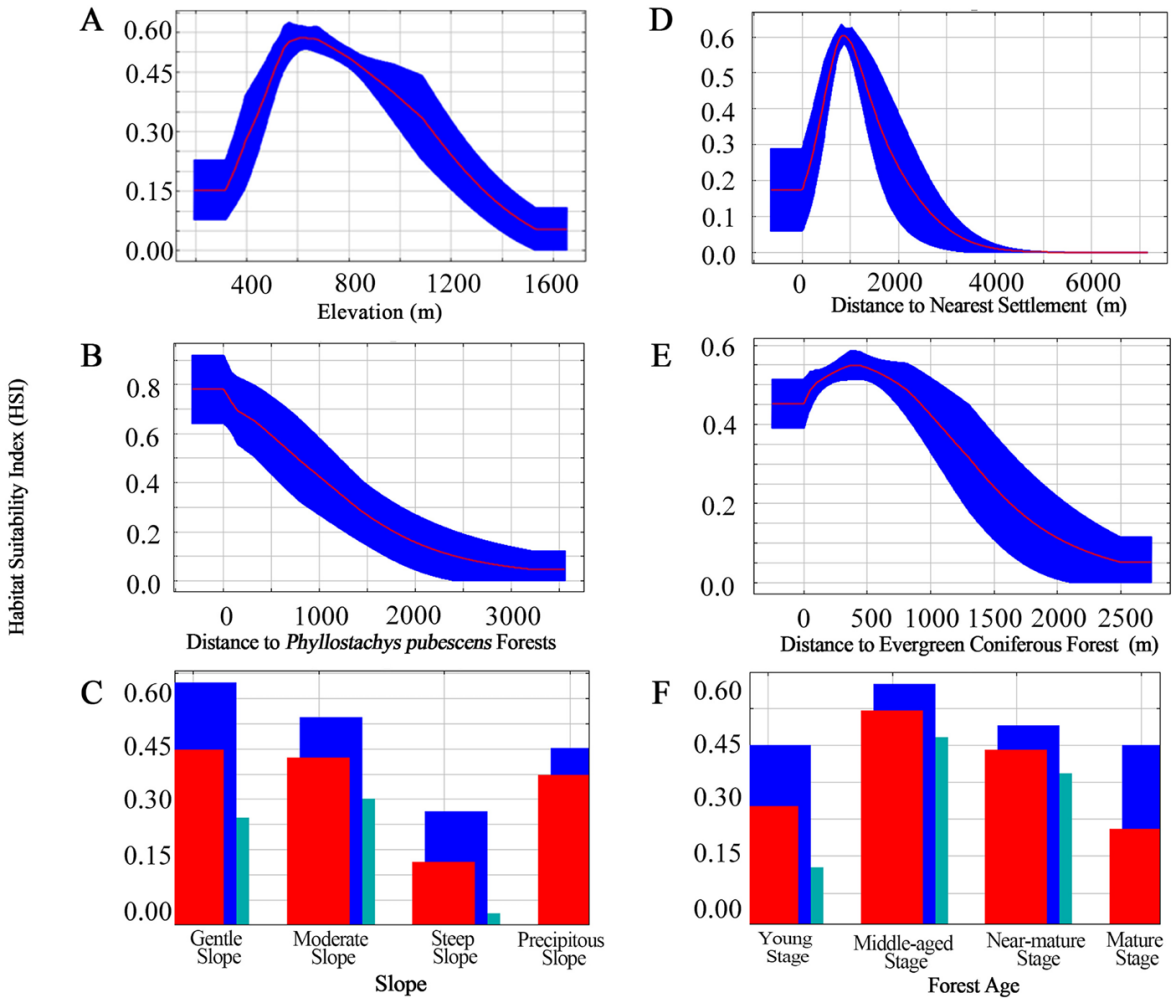
Response curves of key environmental variables affecting habitat suitability of *Sus scrofa* .

For Wild boar, the MaxEnt model also performed well, with a mean training AUC of 0.911 and a mean test AUC of 0.909, averaged across replicates (Figure 5C). Among individual predictors, elevation was the most influential variable (17.1%), followed by distance to the nearest settlement (11.9%) and key vegetation‐related variables, particularly proximity to *Phyllostachys pubescens* forests (9.5%) and evergreen coniferous forests (9.3%) (Table 4). At the categorical level, vegetation and topography together dominated model explanatory power, whereas soil, water‐related, and anthropogenic variables played secondary roles. Response curves (Figure 8) showed that Wild boar habitat suitability peaked at mid‐ to low elevations (around 621 m) and increased with distance from settlements, indicating avoidance of intense human disturbance. The species exhibited strong associations with bamboo forests and forest‐edge environments, reflecting an opportunistic strategy and broad ecological tolerance. Overall, the spatial distribution of Wild boar was characterized by broad ecological tolerance, flexible habitat use, and an opportunistic strategy strongly linked to forest structure and edge habitats.

**TABLE 4:** Relative contributions of environmental variables to habitat suitability for *Sus scrofa*.

Variables	Contributions (%)	Importance (%)
DEM (m)	17.1	22.5
Distance to the Nearest Town (m)	11.9	18.7
Distance to the *Phyllostachys pubescens* (m)	9.5	9.6
Distance to the Evergreen Coniferous Forest (m)	9.3	12.9
Slope (°)	7.4	1.8
Forest age	7.4	1.8
Distance to the Nearest Water (m)	6.5	6.7
Distance to the Evergreen Broad‐leaved Forest (m)	5.4	3.5
Distance to the Evergreen and Deciduous Broad‐leaved Mixed Forest (m)	3.9	3.5
Soil depth (cm)	3.8	4.2
Distance to the Nearest Road (m)	3.4	3.4
Thickness of humus layer (cm)	3.4	1.4
Soil type	3.3	1.4
Total vegetation coverage (%)	2.4	2.3
Distance to the Coniferous and Broad‐leaved Mixed Forest (m)	2.3	3
Aspect	2	1.2
Canopy density	1	0.8

Taken together, the three ungulates exhibited clear interspecific differences in environmental responses and dominant habitat drivers. Black muntjac showed strong specialization associated with water availability and forest concealment, Reeves's muntjac demonstrated broad tolerance to anthropogenic gradients while remaining forest‐dependent, and Wild boar displayed the highest ecological plasticity, particularly in relation to vegetation structure and edge habitats. These contrasting response patterns provide quantitative evidence of spatial niche differentiation among sympatric ungulates.

### 3.2 Spatial Distribution of Suitable Habitats

**FIGURE 9:**
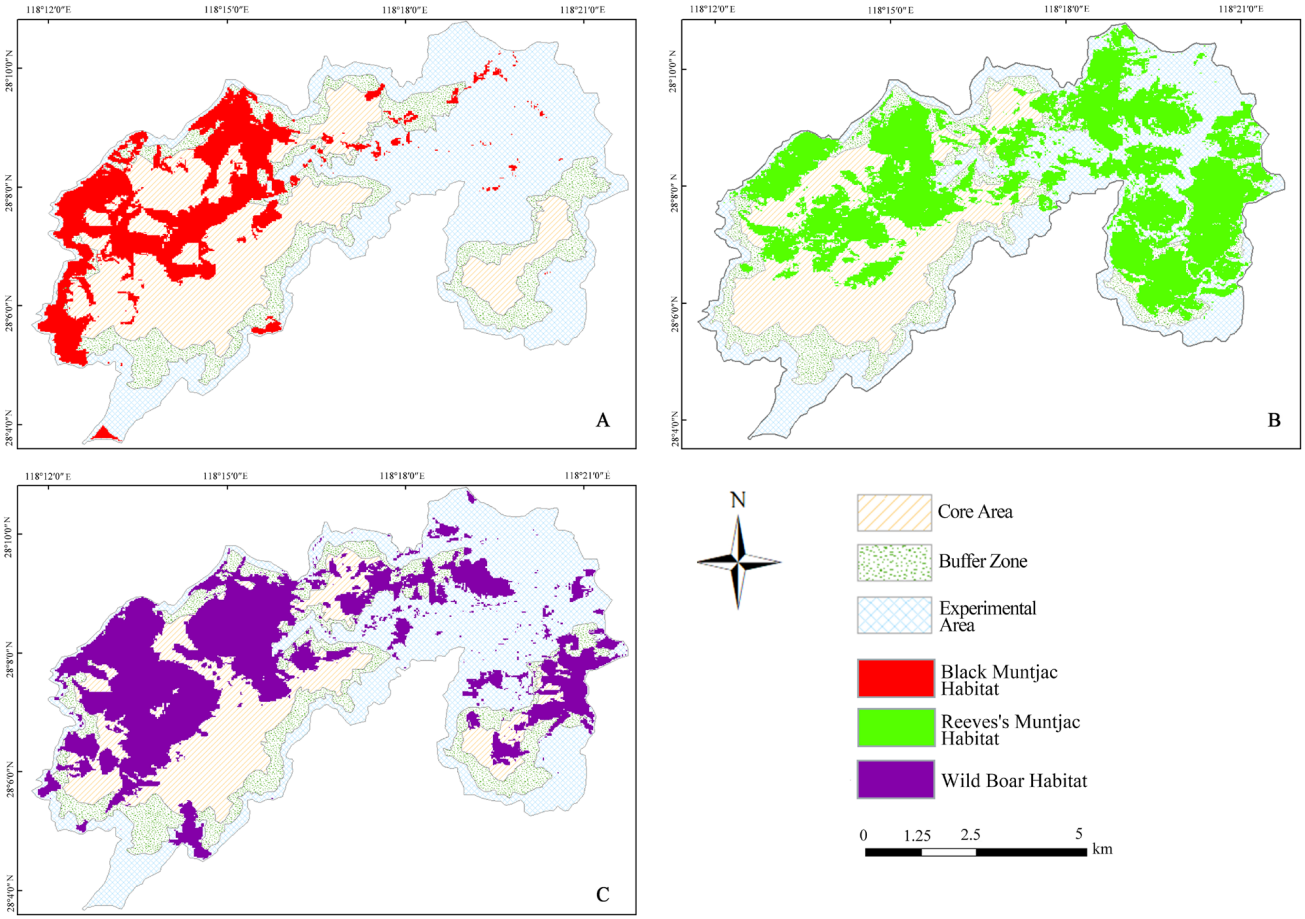
Predicted spatial distribution of suitable habitats for the three ungulate species: (A) *Muntiacus crinifrons* , (B) *Muntiacus reevesi* , and (C) *Sus scrofa* .

According to the MaxEnt model predictions, the mean habitat suitability index (HSI) value across the 10 replicate runs was 0.1568, which was used as a classification threshold. Using this threshold, predicted habitat suitability was reclassified into suitable (HSI ≥ 0.1568) and unsuitable (HSI < 0.1568) areas, yielding spatial distribution maps for Black muntjac, Reeves's muntjac, and Wild boar within the study region (Figure 9).

For Black muntjac, suitable habitats were primarily concentrated in the eastern, central, and northeastern portions of the reserve, forming fragmented and spatially constrained patches with relatively low connectivity (Figure 9A). The total area of suitable habitat was 18.17 km^2^, accounting for approximately 16.8% of the reserve, representing the smallest suitable habitat extent among the three ungulates. Most suitable habitats for Black muntjac were located within the core zone, indicating a strong dependence on well‐protected forest environments. This spatial configuration reflects a narrow niche strategy and high sensitivity to human disturbance, consistent with the species' reliance on rugged terrain and well‐concealed primary forests.

Reeves's muntjac exhibited the widest spatial distribution among the three ungulate species, with suitable habitats extending across the eastern, north‐central, and western parts of the reserve and forming a nearly continuous habitat network (Figure 9B). The total area of suitable habitat reached 45.07 km^2^, accounting for approximately 41.5% of the reserve, representing the largest suitable area among the three species. Suitable habitats of Reeves's muntjac were well represented across all functional zones, with extensive coverage in the core zone and substantial representation in both buffer and experimental zones. This spatial pattern reflects a broad‐niche strategy and high ecological flexibility, enabling the species to exploit diverse forest types and persist under moderate levels of anthropogenic disturbance. As a result, Reeves's muntjac constitutes a typical widespread and ecologically resilient ungulate within the reserve.

For Wild boar, suitable habitats exhibited a multi‐centered and patchy spatial distribution, primarily concentrated in the eastern, northeastern, and western sectors of the reserve (Figure 9C). The total area of suitable habitat was 33.82 km^2^, accounting for approximately 31% of the reserve, indicating a relatively broad spatial extent compared with Black muntjac but smaller than that of Reeves's muntjac. Most suitable habitats were located within the core zone, which alone supported more than half of the predicted suitable area, while the remaining habitats were distributed across the buffer and experimental zones. This spatial configuration suggests a high tolerance to environmental heterogeneity and human disturbance. Wild boar frequently occupied edge environments, particularly bamboo forests and coniferous forest interfaces, reflecting an ecological strategy characterized by broad habitat tolerance coupled with edge‐oriented space use.

### 3.3 Habitat Overlap and Niche Differentiation

**FIGURE 10:**
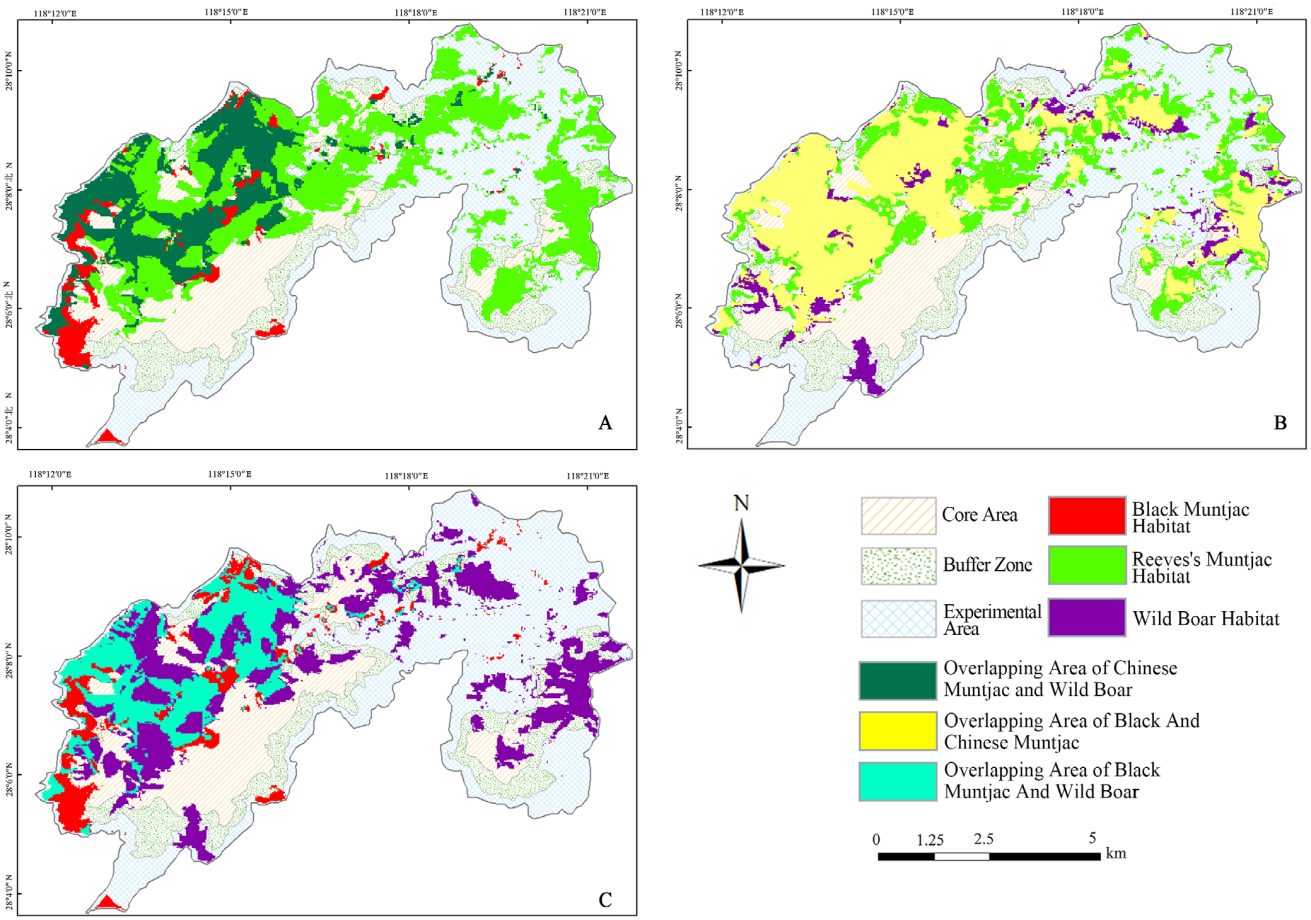
Spatial overlap of suitable habitats among the three ungulate species in the reserve.

Based on MaxEnt predictions and ENMTools analyses, the three ungulate species exhibited pronounced spatial heterogeneity in potential habitat use, reflecting clear patterns of niche differentiation (Table 5; Figure 10). Pairwise niche overlap indices revealed substantial variation in the degree of spatial congruence among species, closely linked to differences in habitat breadth and adaptability. Among the three ungulates, Reeves's muntjac exhibited the broadest spatial distribution and the highest degree of habitat overlap with the other two species. Its overlap with Wild boar was particularly pronounced, with high niche overlap indices (*D* = 0.822, *I* = 0.969) and a shared suitable habitat area of 29.04 km^2^, accounting for 26.77% of the reserve (Figure 10B). This pattern indicates strong spatial congruence, with much of the suitable habitat predicted for Wild boar being encompassed within the broader distribution of Reeves's muntjac, suggesting substantial potential for spatial interaction between these two generalist species.

**TABLE 5:** Pairwise habitat overlap indices among the three ungulate species in the reserve.

Species	Black muntjac	Reeves's muntjac	Wild boar
Black muntjac	1	0.852	0.821
Reeves's muntjac	0.599	1	0.969
Wild boar	0.545	0.822	1

*Note:* The upper triangle shows Hellinger's I, while the lower triangle shows Schoener's D.

In comparison, the spatial overlap between Black muntjac and Reeves's muntjac was intermediate (*D* = 0.599, *I* = 0.852), with a shared suitable habitat area of 14.31 km^2^ (13.19% of the reserve) (Figure 10A). Although portions of suitable habitat overlapped, the core areas of high suitability for the two species showed limited coincidence, indicating partial spatial segregation that may facilitate coexistence through differential habitat use. The lowest degree of overlap was observed between Black muntjac and Wild boar (*D* = 0.545, *I* = 0.821), with only 12.58 km^2^ of shared suitable habitat (11.6% of the reserve) (Figure 10C). Their predicted distributions were largely spatially segregated, reflecting pronounced differences in habitat preferences and limited spatial congruence.

Overall, niche overlap and spatial overlay analyses revealed a clear hierarchical pattern of spatial differentiation among the three ungulates. Reeves's muntjac occupied the broadest niche space and overlapped extensively with both Wild boar and Black muntjac, whereas Black muntjac showed the most restricted distribution and the lowest overlap, particularly with Wild boar. This gradient underscores species‐specific spatial strategies and provides quantitative support for spatial niche differentiation as a key mechanism enabling coexistence in a geographically constrained subtropical mountain landscape.

## 4 Discussion

### 4.1 Environmental Drivers and Niche Differentiation Among the Three Ungulate Species

The formation of habitat suitability is a complex ecological process jointly driven by environmental gradients and species‐specific biological traits (Braunisch et al. 2008). It is not only constrained by static environmental structures but also reflects the capacity of species to dynamically adapt to spatial heterogeneity (Fukuda et al. 2015). Within the framework of resource selection theory (Johnson et al. 2006), food availability, water accessibility, and concealment conditions represent the core ecological axes that shape habitat selection in ungulates, with their spatial configuration exerting a decisive influence on species distributions. Moreover, environmental factors rarely act in isolation; instead, they operate through synergistic mechanisms that collectively structure ecosystems and determine species persistence (Zhang et al. 2020).

Focusing on three representative ungulate species (Black muntjac, Reeves's muntjac, and Wild boar) in Tongboshan National Nature Reserve, our results demonstrate distinct patterns of environmental selection, highlighting the role of environmental filtering under spatial heterogeneity and the influence of long‐term interspecific competition in driving niche differentiation (Hui et al. 2004). MaxEnt model evaluations indicated that the relative importance of predictor variables followed the order: vegetation > topography > water sources and anthropogenic disturbance > soil. This suggests that vegetation structure and habitat accessibility exert the strongest influence on the spatial patterns of ungulates. Such findings not only underscore the direct role of vegetation in providing food and cover but also reflect the complex feedback interactions among variables (Van Breugel et al. 2024). For instance, elevation acts as an integrative factor, directly regulating thermal and hydrological regimes while indirectly shaping vegetation composition, structural layers, and forest succession, thereby influencing animal distribution at multiple levels. These coupled mechanisms reveal the systemic nature of ecosystems and their profound role in sustaining species distribution stability.

Further comparisons indicated that divergence in environmental response patterns underpins spatial niche segregation among the three species. Black muntjac exhibited a relatively specialized ecological strategy, characterized by preferences for higher elevations, steeper slopes, reduced proximity to water, and low levels of human disturbance. As a medium‐sized forest ungulate, it tends to select habitats with high forest connectivity and structurally complex vegetation, likely enhancing concealment and habitat security. This pattern is consistent with findings from neighboring regions and aligns with Hutchinson's niche hypervolume framework, whereby species with narrower niche breadth are more strongly constrained by environmental factors (Chen et al. 2012; Blonder 2018; Zhang et al. 2024; Zheng et al. 2023).

In contrast to the Black muntjac, both Reeves's muntjac and Wild boar exhibited broader niche breadths and greater ecological flexibility. Reeves's muntjac primarily occupied mid‐elevation habitats around 688 m and areas approximately 850 m from human settlements, showing a preference for evergreen coniferous forests and sites close to water sources, while demonstrating moderate tolerance to anthropogenic disturbance. Wild boar, by contrast, displayed the traits of a classic ecological “opportunist” (Schley and Roper 2003), occupying a wide range of suitable habitats and exhibiting strong adaptability to slope gradients, forest types, and human‐modified landscapes. Such generalist strategies not only enhance their spatial expansion potential but also increase resilience and stability in the face of ecosystem disturbances.

From the perspective of spatial nesting, the Black muntjac's niche was situated at the periphery of the broader niches of Reeves's muntjac and Wild boar. Although restricted to a narrower distribution, Black muntjac effectively minimized direct competition with generalist species by selecting highly concealed and low‐disturbance microhabitats. This “niche compression–specialization” strategy represents a key mechanism by which small‐ and medium‐sized ungulates persist in resource‐limited and heterogeneous ecosystems (Bukombe et al. 2018; Trappes 2021). The pronounced elevational gradient and diverse geomorphology of Tongboshan National Nature Reserve provide a spatial template for niche segregation: Black muntjac primarily occupy higher elevations with steep slopes and closed‐canopy forests, whereas Reeves's muntjac and Wild boar are more frequently associated with resource‐rich, open habitats at lower to mid elevations. At the microhabitat scale, species‐specific preferences for vegetation types were also evident. Black muntjac tended to avoid evergreen broadleaf forests and *Phyllostachys pubescens* forests, showing stronger associations with coniferous and broad‐leaved mixed forest. Reeves's muntjac was more frequently distributed in evergreen coniferous forests, while Wild boar was commonly found along forest edges and bamboo–conifer interfaces. This fine‐scale spatial segregation not only reduces the risk of interspecific resource overlap but also reflects the role of the “microhabitat partitioning mechanism” in facilitating species coexistence (Carvajal‐Cogollo and Urbina‐Cardona 2015; Reyes‐Puig et al. 2025).

Despite the high degree of overlap in environmental responses between Reeves's muntjac and Wild boar, indicating substantial potential for spatial interaction, long‐term monitoring suggests that both species coexist extensively within the same landscapes. This coexistence may reflect functional differentiation along non‐spatial niche dimensions; however, such mechanisms remain speculative in the absence of direct temporal or trophic data. Notably, camera‐trap records in this study include precise time stamps, and a preliminary inspection of detection times suggests partial temporal segregation between Reeves's muntjac and Wild boar, with differences in peak activity periods. Differences in diel activity rhythms have been proposed as a common strategy by which sympatric species mitigate overlap (Halle 2000), and our exploratory observations provide initial qualitative support for this possibility. However, this pattern should be regarded as hypothesis‐generating rather than confirmatory, given that no formal temporal niche analysis was conducted in the present study. Accordingly, future studies integrating camera‐trap–derived temporal data with dietary and stable isotope analyses are needed to disentangle temporal and trophic niche partitioning and to clarify the mechanisms underlying multispecies coexistence in heterogeneous landscapes.

We acknowledge that MaxEnt models based primarily on environmental surrogates may overestimate suitable areas, particularly in the absence of fine‐scale field data on forage availability and species‐specific water use. However, in mountainous protected areas, where direct measurements of food resources and water intake are logistically constrained, vegetation structure and distance‐to‐water variables are widely accepted proxies for food and water availability in habitat suitability modeling. Therefore, the predicted habitat suitability in this study should be interpreted as relative suitability patterns rather than absolute occupancy probabilities. Despite these limitations, such relative predictions remain robust and informative for identifying priority conservation areas and guiding spatial management decisions.

### 4.2 Habitat Distribution, Spatial Overlap, and Conservation Implications

Nature reserves play a pivotal role in maintaining regional biodiversity and promoting the long‐term coexistence of multiple species. Based on MaxEnt modeling, this study predicted the potential suitable habitats of three representative ungulates in Tongboshan National Nature Reserve. Results indicate that all three species maintain extensive suitable areas within the reserve, with habitat suitability in the core zone significantly higher than in the buffer and experimental zones. This highlights the effectiveness of the current zoning strategy in safeguarding key habitats. Notably, Reeves's muntjac exhibited the largest suitable area in both the core and experimental zones, reflecting its broad ecological niche and capacity to adapt to diverse environmental conditions.

Although the three species exhibited substantial spatial overlap, clear trends of ecological niche differentiation were evident. Model outputs showed that Black muntjac and Reeves's muntjac shared approximately 14.31 km^2^ of suitable habitat (13.19% of the reserve), primarily concentrated in the northeastern sector. These overlap zones correspond closely with field survey records of frequent muntjac activity, indirectly validating the predictive reliability and applied potential of the models. Importantly, spatial overlap does not necessarily imply direct resource competition. In heterogeneous environments where behavioral strategies differ among species, overlap zones may serve as areas of functional complementarity, fostering “coexistence–synergy” relationships that enhance ecosystem stability (Badali and Zilman 2020; Belso‐Martínez et al. 2017). Similar mechanisms have been reported elsewhere, for instance, zebra ( *Equus quagga* ) and wildebeest ( *Connochaetes taurinus* ) in Kruger National Park coexist despite high dietary overlap by partitioning foraging times and grass‐layer utilization (Arsenault and Owen‐Smith 2002). Likewise, Bawean deer ( *Axis kuhlii* ) and southern red muntjac (*Muntiacus vaginalis*) in Bornean forests exhibit a “core–periphery” nested distribution, facilitating niche differentiation (Rahman and Mardiastuti 2021).

**FIGURE 11:**
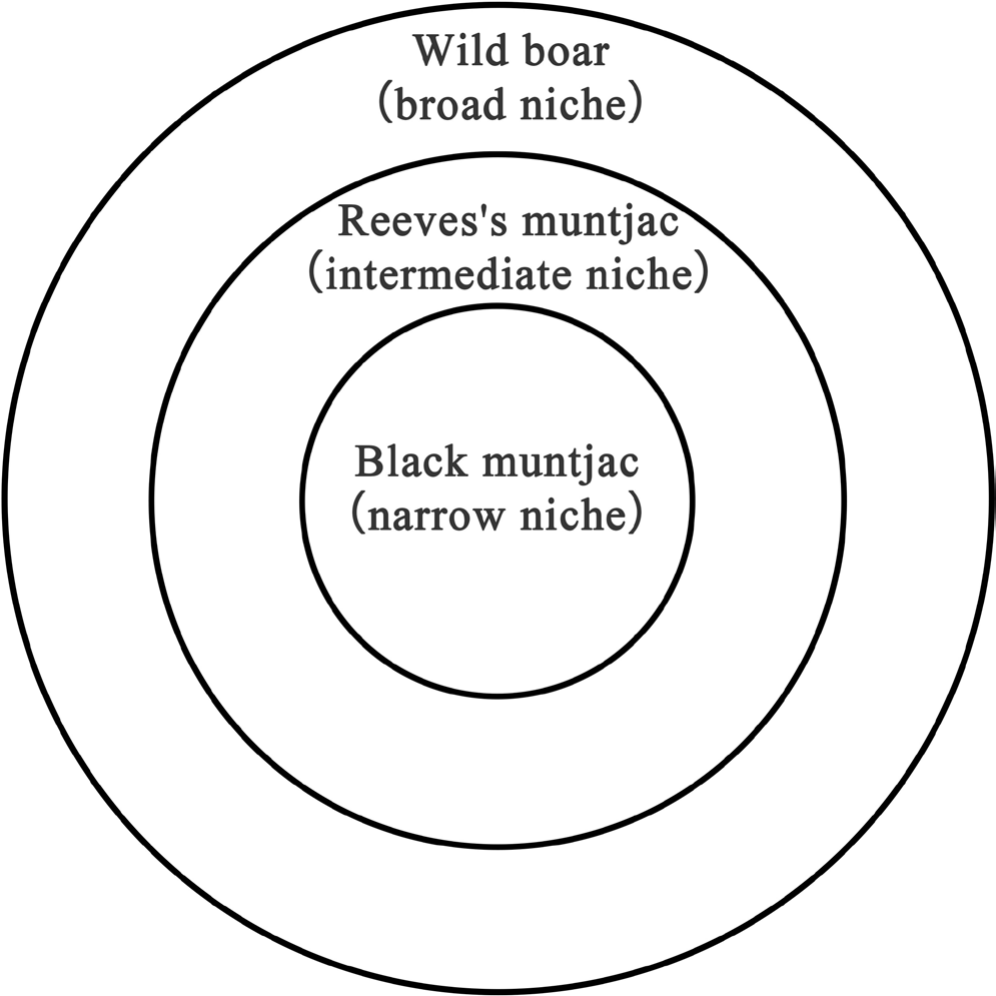
Conceptual diagram illustrating the broad–intermediate–narrow nested hierarchy of suitable habitat niches among Wild boar, Reeves's muntjac, and Black muntjac.

In terms of spatial structure, Reeves's muntjac encompassed the widest range, effectively overlapping nearly the entire distribution of Wild boar and much of that of Black muntjac. This pattern formed a distinct “broad–intermediate–narrow” nested hierarchy (Cutler 2001; Legendre and Fortin 1989), schematically illustrated in Figure 11 (Sugihara et al. 2003; Lu et al. 2025). Wild boar, as an ecological opportunist, demonstrated high plasticity in diet, activity rhythms, and environmental tolerance, allowing it to persist across highly overlapping habitats. By contrast, Black muntjac relied on high‐quality forest microhabitats, preferentially occupying concealed and continuous habitats that reduce spatial overlap with other species. This form of spatial segregation has been documented in other ungulate systems, such as caribou ( *Rangifer tarandus* ) and moose ( *Alces alces* ) in North America, where temporal niche partitioning reduces competition within overlapping ranges (Jung et al. 2015; Leech et al. 2017).

Based on these ecological niche patterns and distributional characteristics, we propose the following conservation recommendations, organized by management priority and explicitly linked to species‐specific results. First, given that Black muntjac exhibited the narrowest spatial niche and the smallest extent of suitable habitat, with strong dependence on high‐elevation, closed‐canopy forests, conservation efforts should prioritize the protection of its core habitats identified by the MaxEnt models. Maintaining habitat closure and integrity in these areas requires continuous enclosure, strict restrictions on mountain access routes, and the prohibition of understory harvesting, directly addressing the species' high sensitivity to disturbance revealed by its niche specialization.

Second, in regions where suitable habitats of Reeves's muntjac and Wild boar show high spatial overlap, particularly along evergreen coniferous forest edges identified as shared suitable areas, management should focus on enhancing vegetation heterogeneity. Restoring multilayered forest structures and establishing mixed tree–shrub–grass belts can increase resource stratification and help alleviate the spatial interaction pressure inferred from the high niche overlap indices between these two species. Third, considering the fragmented distribution of suitable habitats for Black muntjac and the patchy yet extensive distributions of Reeves's muntjac and Wild boar, landscape‐level planning should emphasize the establishment of ecological corridors and auxiliary core habitat belts in buffer and experimental zones. These measures can improve ecological permeability and functional connectivity among habitat patches, as indicated by the spatial configuration of predicted suitable habitats. Finally, to support long‐term conservation effectiveness, a dynamic ecological monitoring platform integrating multi‐scale and multi‐source data should be established. The integration of high‐resolution remote sensing, camera trapping, and DNA fecal sampling, together with spatial prioritization models, can facilitate a “monitoring–modeling–assessment–feedback” adaptive management framework that directly builds on the spatial predictions and niche analyses presented here, supporting evidence‐based decision‐making in protected areas.

## 5 Conclusion

Based on long‐term camera‐trap monitoring in Tongboshan National Nature Reserve, this study integrated MaxEnt species distribution modeling with spatial niche overlap analysis to investigate habitat suitability patterns and coexistence mechanisms among three sympatric ungulates: the Black muntjac, Reeves's muntjac, and Wild boar. Our results revealed pronounced interspecific differences in environmental responses, habitat preferences, and spatial use, indicating clear spatial niche differentiation within this mid‐subtropical montane ecosystem.

The Black muntjac exhibited a highly specialized spatial niche, being primarily restricted to high‐elevation, closed‐canopy forests under minimal human disturbance, whereas Reeves's muntjac displayed broad habitat tolerance characteristic of a generalist strategy. Wild boar showed strong ecological plasticity, occupying fragmented and edge‐associated habitats across the reserve. Consequently, spatial niche overlap followed a nested, hierarchical pattern, with Reeves's muntjac overlapping extensively with the other two species, while Black muntjac and Wild boar showed limited overlap. This pattern highlights the importance of spatial partitioning and habitat heterogeneity in facilitating the long‐term coexistence of sympatric ungulates.

Overall, our findings provide quantitative evidence that conserving habitat specialists such as the Black muntjac requires prioritizing the protection of high‐quality core habitats while maintaining landscape connectivity to support broader community‐level stability. More broadly, this study demonstrates that integrating species distribution modeling with spatial niche analysis offers a powerful framework for informing multi‐species conservation planning in complex mountain landscapes. Future conservation efforts will benefit from incorporating dynamic monitoring approaches that link spatial predictions with ecological processes under ongoing environmental change.

## Author Contributions

**Jie Yao:** conceptualization (equal), data curation (equal), formal analysis (equal), investigation (equal), methodology (equal), resources (equal), validation (equal), visualization (equal), writing – original draft (equal), writing – review and editing (equal). **Xueqin Hu:** conceptualization (equal), formal analysis (equal), investigation (equal), methodology (equal), validation (equal), visualization (equal), writing – original draft (equal), writing – review and editing (equal). **Jun Tian:** formal analysis (equal), investigation (equal), resources (equal), validation (equal). **Feiyan Lv:** data curation (equal), formal analysis (equal), investigation (equal), validation (equal). **Ruijie Yang:** data curation (equal), formal analysis (equal), investigation (equal), validation (equal). **Zhiqiang Huang:** conceptualization (equal), data curation (equal), project administration (equal), resources (equal), supervision (equal). **Jiancheng Zhai:** conceptualization (equal), funding acquisition (equal), methodology (equal), project administration (equal), supervision (equal), writing – original draft (equal), writing – review and editing (equal).

## Funding

This work was supported by Genetic Differentiation of Black Muntjac across Geographic Populations and Habitat Prediction under Climate Change Scenarios, DHYC‐2025042. Investigation and Monitoring Project of Rare Animals such as Black muntjac in Jiangxi Tongboshan National Nature Reserve, H202400127. Project on the Investigation of Primate‐Induced Wildlife Damage in Jiangxi Province (Subproject III), H202500128. Scientific Research Startup Fund of East China University of Technology, DHBK2019064.

## Conflicts of Interest

The authors declare no conflicts of interest.

## Data Availability

The data that support the findings of this study are available within the article, and additional data are available from the corresponding author upon reasonable request.
